# The Cricket Paralysis Virus Suppressor Inhibits microRNA Silencing Mediated by the *Drosophila* Argonaute-2 Protein

**DOI:** 10.1371/journal.pone.0120205

**Published:** 2015-03-20

**Authors:** Corinne Besnard-Guérin, Caroline Jacquier, Josette Pidoux, Safia Deddouche, Christophe Antoniewsk

**Affiliations:** Drosophila Genetics and Epigenetics, Institut de Biologie Paris Seine, CNRS UMR7622 & Université Pierre et Marie Curie, 9 quai Saint-Bernard, F75005, Paris, France; French National Center for Scientific Research—Institut de biologie moléculaire et cellulaire, FRANCE

## Abstract

Small RNAs are potent regulators of gene expression. They also act in defense pathways against invading nucleic acids such as transposable elements or viruses. To counteract these defenses, viruses have evolved viral suppressors of RNA silencing (VSRs). Plant viruses encoded VSRs interfere with siRNAs or miRNAs by targeting common mediators of these two pathways. In contrast, VSRs identified in insect viruses to date only interfere with the siRNA pathway whose effector Argonaute protein is Argonaute-2 (Ago-2). Although a majority of *Drosophila* miRNAs exerts their silencing activity through their loading into the Argonaute-1 protein, recent studies highlighted that a fraction of miRNAs can be loaded into Ago-2, thus acting as siRNAs. In light of these recent findings, we re-examined the role of insect VSRs on Ago-2-mediated miRNA silencing in *Drosophila melanogaster*. Using specific reporter systems in cultured Schneider-2 cells and transgenic flies, we showed here that the Cricket Paralysis virus VSR CrPV1-A but not the Flock House virus B2 VSR abolishes silencing by miRNAs loaded into the Ago-2 protein. Thus, our results provide the first evidence that insect VSR have the potential to directly interfere with the miRNA silencing pathway.

## Introduction

RNA interference (RNAi) provides one of the main lines of defense against RNA viruses in plants and invertebrates [1–4]. Recently, RNAi was also shown to play an antiviral role in mouse embryonic stem cells and suckling mice, suggesting that it could also participate in early antiviral defenses in mammals [5,6].

In *Drosophila melanogaster*, viral infection triggers the production of 21 nt small interfering RNAs duplexes (siRNAs) from the processing of viral double-stranded RNA (dsRNA) intermediates by the RNase III enzyme Dicer-2 (Dcr-2). SiRNA duplexes are loaded into the Argonaute-2 (Ago-2) protein complexes, unwounded and one of the strand guides the cleavage of target RNAs by sequence complementarity [7–9]. The importance of RNAi in *Drosophila* antiviral defense is illustrated by the dramatic increase in sensitivity of *dcr-2* and *ago-2* mutants upon viral infections [1,2,4].

To counterattack, viruses encode viral suppressors of RNAi (VSRs) that interfere with host antiviral silencing [3,10,11]. Several plant viruses express VSRs that use a variety of tactics to inhibit both siRNAs and miRNAs silencing pathways by targeting common processing factors [12–14]. In contrast, VSRs identified in insect viruses are described to specifically suppress the siRNA pathway through diverse evolutionarily convergent strategies. For example, the *Flock House virus* (FHV) B2 binds long dsRNAs to inhibit their processing by Dicer proteins [15–17], and sequesters siRNA duplexes to prevent their loading to Argonaute complexes [18–20]. In contrast, the *Cricket Paralysis virus* (CrPV) CrPV-1A directly binds to the *Drosophila* Ago-2 protein and inhibits both siRNA loading and Ago-2 slicing activity [21].

In animals, microRNAs (miRNAs) regulate most cellular processes including development, cell proliferation, differentiation and immune responses [22]. They mediate gene silencing through imperfect sequence complementarity with their target mRNAs [23]. Duplexes of ∼22nt long miRNA/miRNA* molecules derive from stem-loop precursor transcripts through sequential processing by the Drosha/Pasha microprocessor and the Dicer-1/Loqs complex [24]. In *Drosophila melanogaster*, most miRNA duplexes are loaded into a RNA-induced silencing complex (RISC) containing Argonaute-1 (Ago-1). Following elimination of the miRNA* passenger strand, the mature miRNA strand guides Ago-1 for translational repression and/or destabilization of mRNA targets [23]. Pairing between the 5’ seed regions of miRNAs and the 3’UTR of their mRNA targets is generally sufficient to ensure silencing [25–27]. In *Drosophila*, the Ago-2-dependent siRNA and Ago1-dependent miRNA pathways are compartmentalized. Nevertheless, a subset of miRNAs do not conform to this compartmentalization: they preferentially associate with the RNAi component Ago-2 and, similarly to siRNA, can guide potent mRNA target slicing, provided that they have extensive sequence complementarity with the target [9,26,28–30]. The *Drosophila* community largely exploits this miRNA property for gene knockdown experiments using Valium transgenes that express artificial miRNAs reprogrammed against specific gene sequences [31–33]. Likewise, we have recently shown that artificial miRNAs with perfect complementarity to GFP sequences interact with Ago-2 for potent GFP reporter silencing [34].

In light of these recent developments, we decided to re-visit the effects of VSRs on silencing mediated by miRNA associated with Ago-2. Here we show that CrPV-1A but not B2 strongly interfere with the Ago-2-dependent miRNA silencing. Altogether our results demonstrate that VSRs targeting Ago-2 processing complexes have the potential to directly interfere with part of the miRNA regulatory network in *Drosophila*.

## Results

### The CrPV-1A but not the B2 VSR inhibits the Ago-2-dependent *Drosophila* miRNA pathway

**Fig 1:**
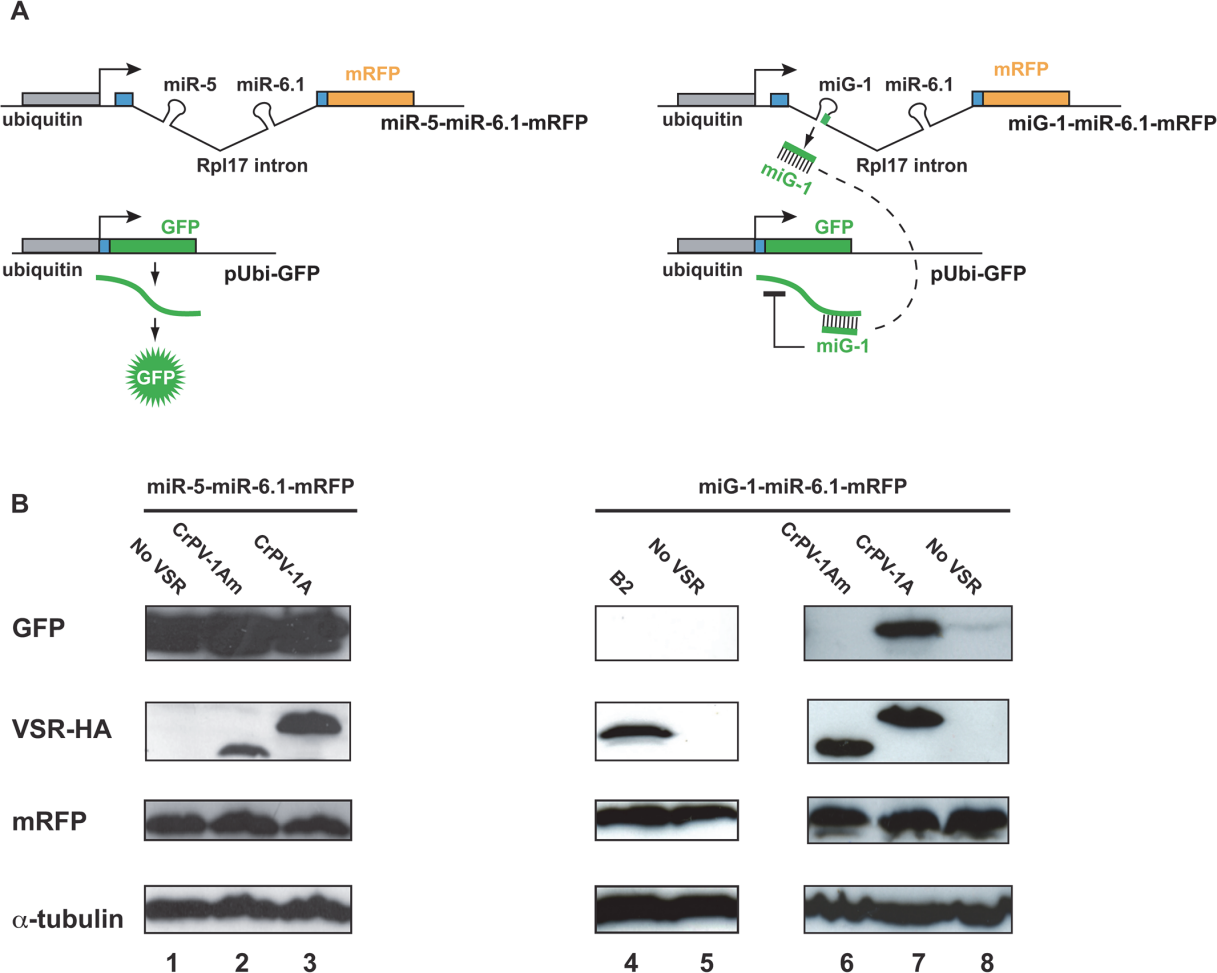
CrPV-1A suppresses the Ago-2-dependent miRNA silencing in S2 cells. (A) Schematic representation of the reporter system for miRNA silencing. Pre-miRNAs inserted in an *Rpl17* intron and the mRFP gene are expressed from the same unspliced transcript under the control of the *ubiquitin* promoter. The miR-5-miR-6.1-mRFP construct (left), expresses miR-5 and miR-6.1 without complementarity to the GFP and was used as a control. The miG-1-miR-6.1–mRFP construct (right) expresses miR-6.1 as well as miG-1 which targets the GFP mRNA expressed from pUbi-GFP with perfect complementarity. (B) Western blot analysis of Drosophila S2 cell lysates, co-transfected with miRNA expression constructs described in (A), the pUbi-GFP sensor plasmid, and the C-terminal HA-tagged CrPV-1A or B2 expression vectors. Control plasmid with non-cognate miR-5 and miR-6 was used as a control for miRNA target specificity and α-tubulin is the loading control. One representative experiment out of five is shown.

In order to analyze the effect of VSRs on miRNAs loaded into Ago-2, we took advantage of the automiG reporter system recently developed in our laboratory [34]. As previously described, this reporter is sensitive to Ago-2-silencing activity mediated by the artificial miRNAs miG-1 and miG-2 targeting GFP sequences. Here, we set up an automiG-derivate system combining a miG-1-miR-6.1-mRFP silencing vector and a pUbi-GFP target plasmid (Fig. 1A). The *ubiquitin* promoter in the miG-1-miR-6.1-mRFP construct drives the expression of both the monomeric Red Fluorescent Protein and the stem-loop precursor sequences of miG-1 and miR-6.1 miRNAs inserted in an Rpl17 intron (Fig. 1A, right panel). It is noteworthy that since mRFP and miG-1 expressions rely on the same *ubiquitin* promoter, the level of mRFP protein directly reflects the expression level of miG-1. As a negative control, we used the miR-5-miR-6.1-mRFP construct that is devoid of GFP-targeting miRNA (Fig. 1A, left panel).

The GFP protein was readily detected in western blot experiments upon co-transfection of *Drosophila* S2 cells with the miR-5-miR-6.1-mRFP control plasmid and the pUbi-GFP target plasmid (Fig. 1B, lane 1). In contrast, the GFP protein was barely detectable in the presence of the miG-1-miR-6.1-mRFP silencing plasmid (Fig. 1B, lanes 5 and 8), indicating that miG-1 efficiently silences GFP expression. The mRFP protein was detected at similar level across samples, which reflected similar levels of miRNA expression (Fig. 1B).

The GFP protein was also undetectable upon co-transfections of miG-1-miR-6.1-mRFP, pUbi-GFP with a vector expressing the HA-tagged B2 VSR (Fig. 1B, compare lane 4 with lane 5). As the HA-tagged B2 protein was shown to efficiently suppress RNAi triggered by long dsRNA in S2 cells (CA, unpublished results), this result indicates that active B2 is unable to suppress miG-1 silencing activity. In striking contrast, strong expression of the GFP protein was restored in the presence of a vector expressing the HA-tagged CrPV-1A VSR (Fig. 1B, lane 7), which was previously characterized as a 148 amino acids (aa) polypeptide able to bind Ago-2 and efficiently suppress its silencing activity [21]. A C-terminal shortened 108 aa CrPV-1Am polypeptide fails to bind Ago-2 and to suppress siRNA silencing [21]. Consistently, a construct expressing this HA-tagged CrPV-1Am mutant form failed to suppress GFP silencing (Fig. 1B, lane 6) indicating that direct interaction with Ago-2 is required. Together, these data indicate that the CrPV-1A VSR efficiently suppress Ago-2-mediated miG-1 silencing.

### The CrPV-1A VSR can prevent the silencing of miRNAs with perfect or imperfect mRNA binding sites

**Fig 2:**
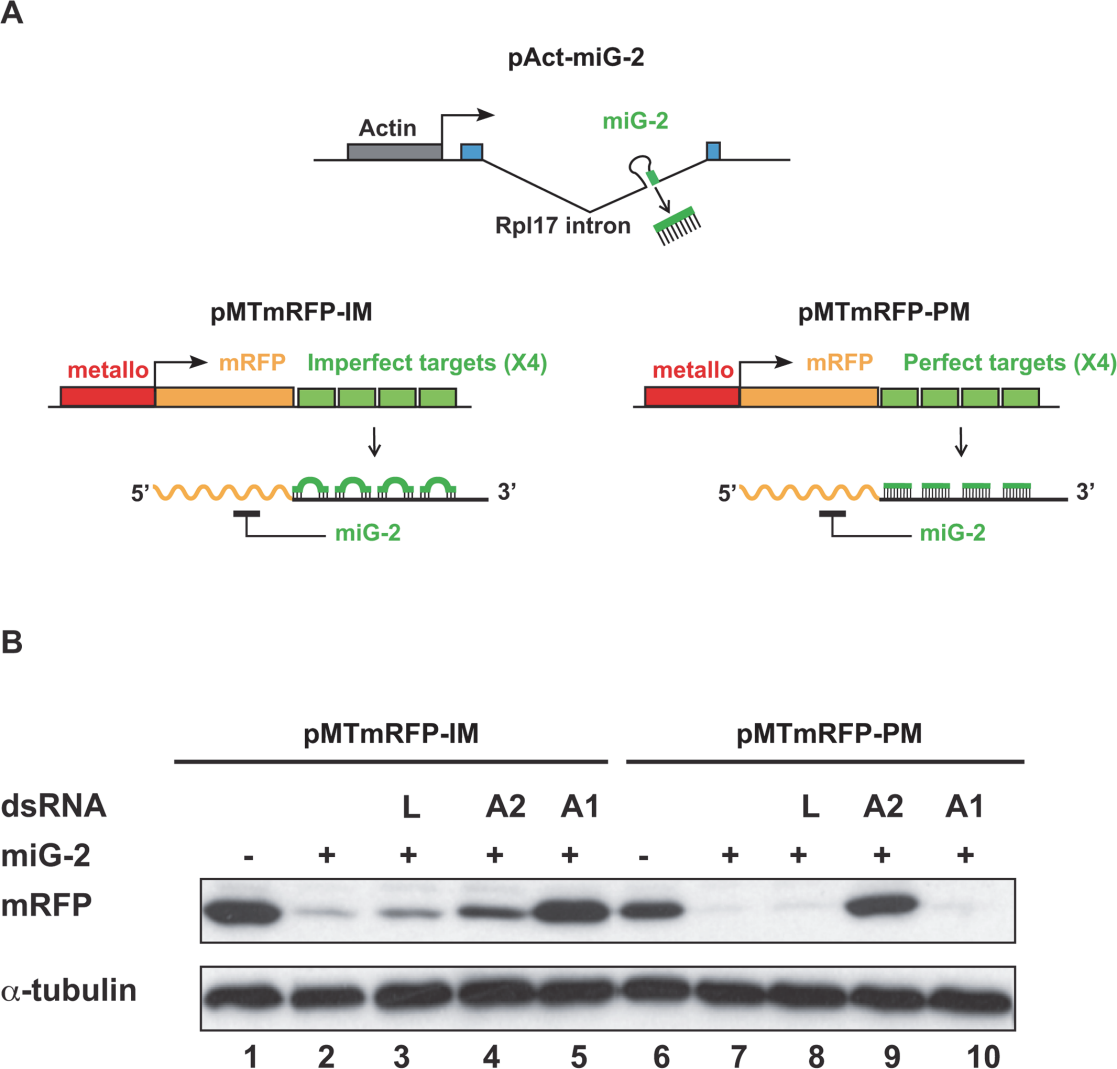
Silencing of mRFP reporters bearing four imperfect or perfect miG-2 binding sites in Drosophila S2 cells. (A) Schematic representation of miG-2 silencing sensors. The copper inducible *metallothionein* promoter drive the expression of the mRFP transcript bearing perfectly matched (pMTmRFP-PM) or imperfectly matched (pMTmRFP-IM) miG-2 target sites. Expression of miG-2 is provided in *trans* by the pAct-miG-2 construct. (B) Western blot analysis of Drosophila S2 cells co-transfected with pAct-miG-2, pMTmRFP-IM or pMTmRFP-PM and the indicated dsRNAs targeting Ago-2 (A2), Ago-1 (A1) or the control luciferase dsRNA (L). α-tubulin is used as loading control. One representative experiment out of four is shown.

Most endogenous miRNAs imperfectly match multiple sites in their mRNA targets [25]. We thus examined the effect of CrPV-1A on miRNAs imperfectly matching their complementary sequences. To this end, we set up two additional reporter systems based on mRFP silencing by the artificial miG-2 miRNA (Fig. 2A), that can be loaded into either Ago-1 or Ago-2 complexes [34]. mRFP target constructs in these systems were generated by inserting 4 target sites with perfect (pMTmRFP-PM) or imperfect (pMTmRFP-IM) matches to miG-2 in the 3’UTR of an mRFP reporter gene driven by the copper-inducible *metallothionein* promoter (Fig. 2A).

In the absence of pAct-miG-2, expression of the mRFP protein was readily detected in transfected cells after induction with copper sulfate of pMTmRFP-IM or pMTmRFP-PM target constructs (Fig. 2B, lanes 1 and 6). As expected, mRFP expression from both constructs was strongly reduced in the presence of the miG-2 expressing vector pAct-miG-2 (Fig. 2B, lanes 2 and 7). To further characterize the miG-2 silencing activity in our reporter system, we carried out knockdown experiments of Ago-1 and Ago-2 as previously described [34]. Thus, Ago-1 or Ago-2 dsRNAs were co-transfected with pMTmRFP-IM or pMTmRFP-PM. The reporter constructs were copper-induced 24 hours post-transfection and mRFP expression was monitored by western blot 48 hours later.

Silencing of pMTmRFP-PM was released upon Ago-2 knockdown but not upon Ago-1 knockdown (Fig. 2B, lanes 9 and 10), indicating that pMTmRFP-PM exclusively reports for miG-2 silencing through Ago-2. In contrast, silencing of pMTmRFP-IM was strongly released upon Ago-1 knockdown and, to a lesser extent, upon Ago-2 knockdown (Fig. 2B, compare lanes 5 and 4 with the luciferase dsRNA control lane 3). Altogether (one representative experiment out of four is shown in Fig. 2B) these results indicate that pMTmRFP-IM is repressed by Ago-1 associated with miG-2, agreeing with the notion that Ago-1 accommodates well imperfectly matched target sites. Nevertheless, our results also indicates that part of the pMTmRFP-IM silencing involves Ago-2 which is consistent with previous reports showing that Ago-2 can mediate silencing through imperfectly matched target sites, although to a lesser extent than through perfectly matched target sites [27].

**Fig 3:**
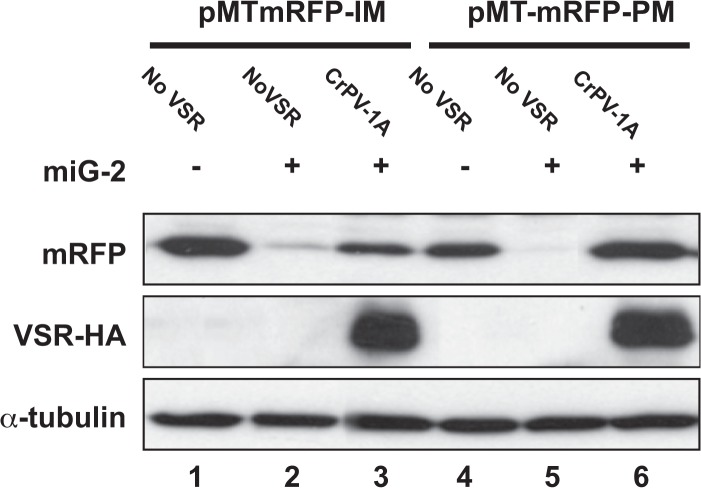
CrPV-1A interferes with miRNA silencing mediated by perfect or imperfect target sites in S2 cells. Western blot analysis of S2 cells co-transfected with pMTmRFP-IM or pMTmRFP-PM, pAct-miG-2 and CrPV-1A-HA or no VSR expression constructs. α-tubulin is shown as loading control. One representative experiment out of four is shown.

We then tested the ability of CrPV-1A to suppress the silencing of the target constructs pMTmRFP-PM and pMTmRFP-IM by miG-2 in S2 cells (Fig. 3, one representative experiment out of four is shown). In the presence of the CrPV-1A-HA expression vector, the silencing of pMTmRFP-PM by miG-2 (compare lanes 4 and 5 in Fig. 3) was completely released (Fig. 3, lane 6). In contrast, the silencing of pMTmRFP-IM was released to a lesser extent (Fig. 3, lanes 1 to 3). These results further support that CrPV-1A suppresses miRNA silencing mediated by the Ago-2 argonaute protein and has little if any effect on Ago-1-mediated miRNA silencing.

### The CrPV-1A VSR interferes with the Ago-2-dependent miRNA pathway in adult flies

**Fig 4:**
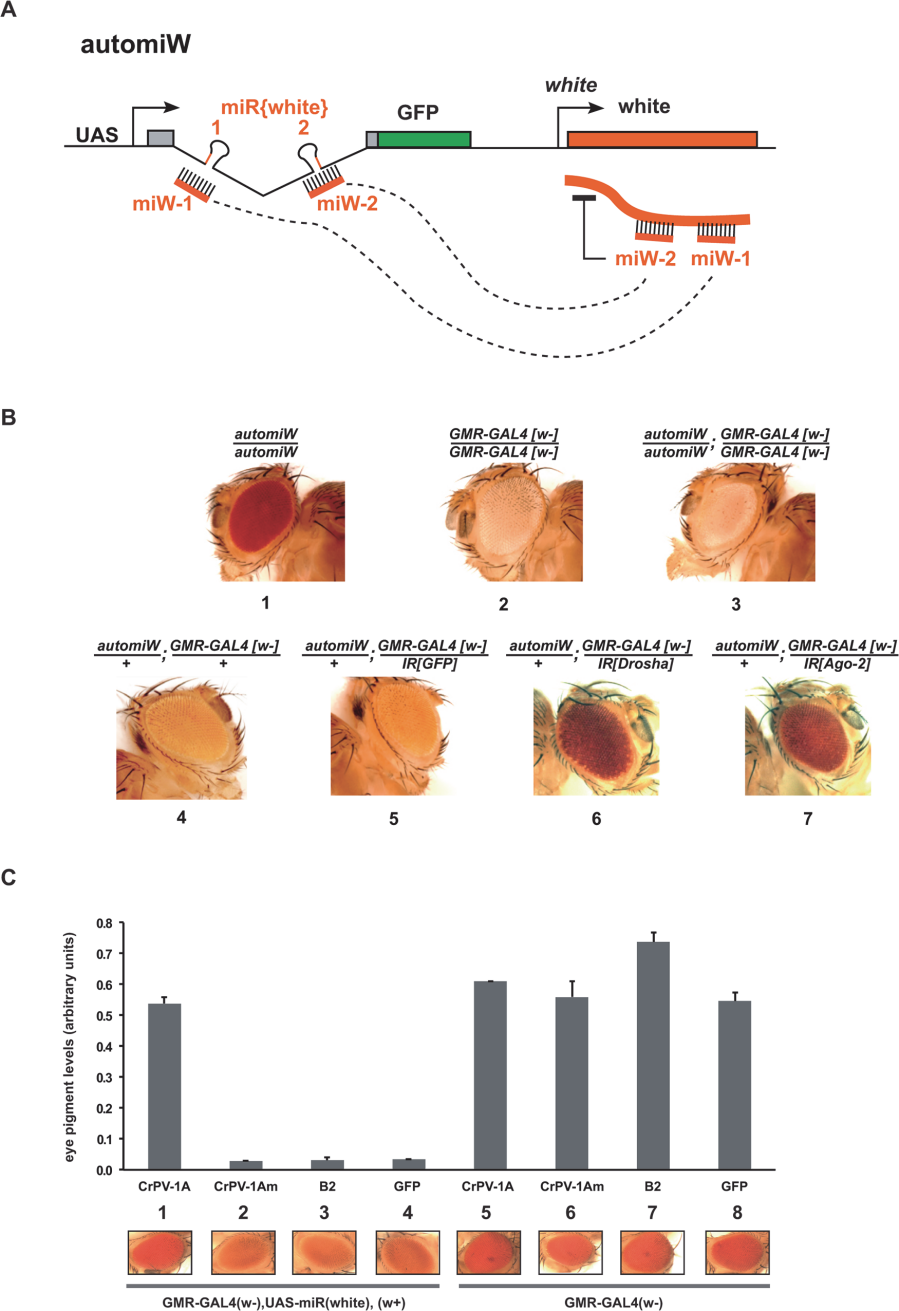
CrPV-1A but not B2 suppresses Ago-2 mediated miRNA silencing in adult flies. (A) Structure of the automiW transgene used to induce *white* silencing in adult eyes. The GMR promoter drives transgene expression of the miW miRNAs together with the white gene in differentiated eye. The two miW-1 and miW-2 will repress expression of the white gene resulting in white colored eyes. (B) Analysis of the automiW silencing in adult fly eyes. Silencing of the *white* maker gene of automiW was triggered in the presence of a GMR-Gal4 transgene and analyzed upon GFP, Drosha or Ago-2 RNAi knockdown by inverted repeats transgenes (IR). Fly genotypes and transgenes dosages are indicated above panels. (C) Analysis of automiW silencing in adult female eyes in the presence of one copy of the GMR-gal4 driver and one copy of the indicated VSR transgene (lane 1 to 3). A UAS-GFP transgene was used a control (lane 4). Note also that in the absence of the automiW transgene in heterozygous combinations of GMR-GAL4 with a UAS-VSR or UAS-GFP (lane 5 to 8), the mini-white markers of the two transgenes produced equivalent strong red eye pigmentation. Upper panel: eye pigmentation content was measured by pigment dosage for flies carrying the indicated transgenes. Histogram shows the mean values and error bars indicated standard deviation for three experiments. Bottom panels: one representative eye image is shown for each genotype.

To further investigate the effect of CrPV-1A on Ago-2 miRNA silencing in flies, we set up a transgenic reporter system. Thus, we established a *Drosophila* transgenic line expressing an automiW construct containing a UAS promoter driving the expression of the GFP protein together with two artificial miRNAs miW-1 and miW-2 embedded in the *Rpl17* intron (Fig. 4A). The sequences of the mature miW-1 and miW-2 miRNAs were designed to be perfectly complementary to sequences of the *white* gene marker in the construct, whose product is involved in red pigment deposition in adult eyes.

In the absence of GAL4 driver, homozygous automiW transgenic flies harbored a dark red eye color (Fig. 4B, panel 1). In contrast, flies homozygous for both the eye-specific GMR-GAL4 [w-] driver which does not bear a functional *white* gene (see http://flybase.org/reports/FBti0072862.html) and the automiW transgenes harbored a white eye color (Fig. 4B, panel 3), similar to that observed in homozygous GMR-GAL4 [w-] flies (Fig. 4B, panel 2). This result indicated that the artificial miW-1 and miW-2 miRNAs silence mini-white expression *in vivo*. Although to a lesser extent, this silencing was still efficient in flies heterozygous for both the GMR-GAL4 [w-] driver and the automiW transgene (compare the white and orange pale colors in Fig. 4B, panels 3 and 4, respectively). This data indicates that the level of white silencing depends on the relative gene dosage of the GAL4 driver and automiW transgene. RNAi knockdowns of Drosha or Ago-2 by inverted repeats (IR) strongly inhibited the *white* gene silencing in these heterozygous GMR-GAL4 [w-], automiW flies (compare Fig. 4B, panels 6 and 7 with the control GFP RNAi knockdown in panel 5). These data strongly suggest that the automiW system is a sensitive biosensor for Ago-2 mediated silencing of the *white* gene by the artificial miW-1 and miW-2 miRNAs.

We then examined the effect of CrPV-1A on the automiW silencing using a UAS-CrPV-1A-Flag transgene [21]. When co-expressed under the control of the eye-specific GMR-GAL4 [w-] driver with the automiW transgene, CrPV-1A but not its mutant version UASp-CrPV-1Am efficiently suppressed the *white* silencing by miW-1 and miW-2 (Fig. 4C, compare panels 1 and 2 with flies expressing a control GFP protein in panel 4). As expected from our previous observations in S2 cells, expression of the B2 VSR did not suppress the silencing of the automiW system (Fig. 4C, panel 3). Altogether, our data indicate that, in addition to its antiviral function, the CrPV-1A VSR can inhibit Ago-2-mediated miRNA silencing in adult flies.

## Discussion

So far, insect VSRs were only shown to interfere with the RNAi pathway. The Flock House virus B2 as well as the *Drosophila* C Virus 1A VSRs interfere with the Dicer-2 processing of long double-stranded RNAs into siRNAs. Several evidence also suggest than B2 sequesters siRNA duplexes, preventing their loading into Ago-2. In contrast, both these VSRs failed to inhibit the silencing of reporter systems by the endogenous miRNAs miR-2b, bantam or miR-7 [35–37]. These observations support the notion that B2 and DCV-1A interfere with siRNA biogenesis but not with miRNA biogenesis.

Recent studies revealed that a fraction of miRNAs mediate silencing of perfectly complementary targets through their loading into Ago-2 [9,26,28–30]. As the Cricket Paralysis virus VSR CrPV-1A was shown to specifically interfere with Ago-2 [21], we decided to analyze its effect on Ago-2-mediated miRNA silencing. Indeed, we have shown here that CrPV-1A is a potent inhibitor of miRNA silencing in both S2 cells and transgenic flies, when this silencing is mediated by Ago-2. In agreement with a previous report [21], CrPV-1A had no noticeable effect on Ago-1-mediated miRNA silencing (see Fig. 3). In contrast, we did not detect any effect of the B2 VSR on Ago-2-mediated miRNA silencing in S2 cells (Fig. 1) or in adult flies (Fig. 4). These results are in agreement with previous findings that B2 interfere with siRNA biogenesis but not with the biogenesis or the silencing activity of miRNAs [15–17,36,38]. Previously, only one report had suggested a potential role of an insect virus VSR on the miRNA pathway in insect cells [39]. Using hairpin construct targeting GFP, Kakumani et al. showed that the Dengue virus NSB4 was able to inhibit miRNA dependent silencing. This is in concordance with our data showing that most of the perfectly complementary miRNA are mediating silencing through Ago-2. It is also interesting to speculate that Dengue NS4B could be interfering at the level of Ago-2 explaining why similarly to CrPV-1A, it inhibits both RNAi and the Ago-2-dependent miRNA pathway.

Transgenic *Drosophila* lines expressing the CrPV-1A suppressor do not exhibit noticeable developmental defects and are viable and fertile. These observations are in agreement with the findings that essential functions fulfilled by miRNAs in development, differentiation and homeostasis are mediated by Ago-1 whose loss-of-function mutations are lethal during early embryogenesis [7]. In contrast, Ago-2 is dispensable under normal laboratory conditions [7] and the biological function of Ago-2 miRNA mediated silencing remains to be established. Bioinformatics analyses have failed to reveal perfectly complementary targets of Ago-2 loaded miRNAs or miRNA*s in the transcriptome [28]. However, the clear biochemical evidence for Ago-2 loading by endogenous miRNA and miRNA*s suggest that this feature is under selection pressure. It is possible that some yet to be identified RNAs with more limited complementarity to miRNAs and miRNA*s are indeed targeted by Ago-2 silencing, either through cleavage or translational repression [27]. The CrPV-1A suppressor would likely affect the regulation of such putative mRNA targets. Interestingly, a recent work suggests a potential role of the miRNA loaded into Ago-2 complexes in aging. It was shown in *Drosophila* that partitioning of miRNAs between Ago-1 and Ago-2 is modulated with age. This shift of miRNAs into Ago-2 complexes is associated with a potential effect on targets downregulation. Furthermore, disruption of this process in *ago-2* mutant is associated with neurodegeneration and reduced life span [40]. Thus, it will be of high interest to test in further studies whether expression of CrPV-1A induces similar phenotypes and has impact on *Drosophila* aging.

CrPV-1A is not the only VSR able to target Ago-2 function. Recently the ORF1 of Nora virus was identified as a potent inhibitor of RNAi by targeting Ago-2 [35]. Importantly, Nora virus is a persistent virus shown to be naturally present in many laboratory and wild-type *Drosophila* strains [41]. If our observation on the effect of CrPV-1A holds true for Nora virus ORF1 suppressor, it is most likely that Nora virus ORF1 will interfere with the Ago-2 dependent miRNA pathway in infected tissues. This is particularly important for *in vivo* gene knockdown experiments that rely on the use of *Drosophila* hairpins to silence perfectly complementary target genes which most likely exert their silencing activity through Ago-2. In light of our results, it is possible that conditions such as persistent Nora virus infections will impair gene knockdown experiments using these tools.

## Materials and Methods

### Plasmid constructs

#### VSR plasmids

VSR cDNAs were cloned into pENTRY-TOPO vector (Life Technologies) as described previously [21,36] and were subsequently transferred using the Gateway system (Life Technologies) into the pAWH destination vector which allows expression of the insert fused to a C-terminal hemagglutinin tag (3xHA) under the control of the constitutive *Actin 5C* promoter.

#### miRNA silencing reporter systems

The pENTRY-miR-5-miR-6.1 and pENTRY-miG-1-miR-6.1 plasmids [34] were recombined with the Gateway pUWR destination vector (a kind gift from Clara Moch and Jean-Rene Huynh) described at the Drosophila Genome Ressource Center (https://dgrc.cgb.indiana.edu/) and carrying an *ubiquitin* promoter and a mRFP gene reporter to generate the miR-5-miR-6.1-mRFP and miG-1-miR-6.1-mRFP plasmids respectively (Fig. 1A).

To generate the pMTmRFP-PM and pMTmRFP-IM sensor plasmids, we first PCR-amplified a fragment carrying mRFP coding sequences using the primers NotI- ATG-mRFP and XhoI-Stop-mRFP (S1 Table). The resulting mRFP DNA fragment was inserted between the NotI and XhoI restriction sites in place of the GFP sequences in the pENTRY-miG-1-miG-2-GFP plasmid [34] to generate the pENTRY-miG-1-miG-2-mRFP plasmid. DNA oligonucleotides (S1 Table) containing four sites complementary to the miG-2 mature sequence without (PM) or with (IM) mismatches at positions 9–11 relative to the 5’ end of miG-2 were annealed and cloned into pENTRY/D-TOPO and next subcloned into the pENTRY-miG-1-miG-2-mRFP-stop plasmid using XhoI and XbaI sites. The pENTRY-miG-1-miG-2-mRFP-PM and pENTRY-miG-1-miG-2-mRFP-IM plasmids were then deleted of the miG-1 and miG-2 sequences by digestion/re-ligation with EcoRI/SphI and HindIII/ClaI, respectively. Finally, the resulting pENTRY-mRFP-PM and mRFP-IM constructs were recombined with the pMT-DEST48-V5-His vector (Life Technologies), generating the pMTmRFP-PM and pMTmRFP-IM biosensors inducible by copper.

To generate the pAct-miG-2 plasmid, we deleted the pre-miG-1 sequences from the pENTRY-3C_miG-1_miG-2 plasmid [34] by EcoRI/SphI digestion and re-ligation. The resulting pENTRY-3C_miG-2 construct was then recombined with pAWH destination vector to give pAct-miG-2.

To generate the automiW construct, DNA oligonucleotides containing the backbone sequences of pre-miR-5 and pre-miR6-1 [34] and 22 nt perfectly complementary to exons 6 and 5 of the white gene, respectively, in place of the mature miR-5 and miR-6.1 sequences (bolded in S1 Table) were annealed and cloned in place of miG-1 and miG-2, respectively, in the pENTRY-3C_miG-1_miG-2 construct [34]. The resulting pENTRY-3C_miW-1_miW-2-GFP was then transferred using the Gateway technology (Life Technologies) into pTGW (Drosophila Gateway Vector collection, Carnegie Institution) to generate the automiW construct which contains an attB site pour PhiC31 mediated transgenesis as well as a mini-white gene transformation marker (Fig. 4A).

### Cells

Drosophila S2 cells (Invitrogen) were cultured at 25° C in Schneider’s Drosophila medium (GIBCO) supplemented with 5% fetal calf serum, 100 U/ml penicillin and 100 ug/ml streptomycin. S2 cells were seeded at 2.5 x10^4^ cells per well of 24-well plates and grown for 24 hours before transfection. Transient transfections were performed using Effectene reagent (QIAGEN) according to manufacturer’s instructions. Ago-1, Ago-2 and luciferase double-stranded RNAs were generated as described in Carré et al., 2013. For RNAi knockdown experiments, 3 μg of dsRNA were co-transfected with the appropriate DNA plasmids using Effectene reagent (QIAGEN) and expression of the reporter plasmids was triggered 24 hours later by addition of 500 μM CuSO4. After 48 additional hours, cells were harvested for western blot analyses.

### Western-blot analysis

For protein analysis, equal amounts of proteins extracted from transfected S2 cells were denatured in Laemmli sample buffer, submitted to 12% SDS-PAGE and transfered onto nitrocellulose membranes. After blocking for one hour in PBS supplemented with 0.1% Tween 20 (TPBS) and 5% fat-free milk, membranes were incubated in TPBS overnight at 4°C in the presence of appropriate primary antibodies. Antibodies were obtained from the following sources: mouse anti-GFP (Roche), rabbit anti-mRFP polyclonal rabbit (Clontech), mouse anti-tubulin (Tebu santa cruz), rat anti-HA (Roche), mouse anti-luciferase (Sigma). After three washes in TPBS, the membranes were incubated for 2H at room temperature in TPBS supplemented with 5% fat-free milk and HRP-conjugated secondary antibodies from Amersham. After three washes in TPBS, detection was performed using ECL Western Blotting (Pierce).

### Transgenic lines

Drosophila transgenic lines for the UASp-CrPV-1A, UASp-CrPV-1Am, UASp-B2 and UASp-GFP constructs were established previously [36]. The transgenic automiW was established using PhiC31 integrase mediated transgenesis [42] of the strain 24483 (Bestgene Inc) which contains an attP docking site at 51D9 in the right arm of chromosome 2.

### Eye pigment dosage

Assays were performed as previously described [36] on one day old virgin females. Five heads per genotype were manually collected and homogenized in 50 μl of a freshly prepared solution of acidified methanol (0.1% HCl). Pigment was extracted by rocking tubes for 36 hours at 4°C. Homogenates were then incubated at 50°C for 5 min, clarified by centrifugation and optical density of each sample was read at 480 nm. Three independent extractions were performed for each genotype and the mean values of the absorption per extraction were calculated.

## Supporting Information

S1 Table Primers and oligonucleotide sequences. Sequences of mature miRNAs are bolded.
(DOCX)
