# Correction: The Cricket Paralysis Virus Suppressor Inhibits microRNA Silencing Mediated by the *Drosophila* Argonaute-2 Protein

**DOI:** 10.1371/journal.pone.0126055

**Published:** 2015-05-06

**Authors:** The PLOS ONE Staff

The fifth author’s name is spelled incorrectly. The correct name is: Christophe Antoniewski. The complete, correct citation is as follows:

Besnard-Guérin C, Jacquier C, Pidoux J, Deddouche S, Antoniewski C (2015) The Cricket Paralysis Virus Suppressor Inhibits microRNA Silencing Mediated by the *Drosophila* Argonaute-2 Protein. PLoS ONE 10(3): e0120205. doi:10.1371/journal.pone.0120205

The publisher apologizes for the error.
