# Supplementary material for: The Cricket Paralysis Virus Suppressor Inhibits microRNA Silencing Mediated by the Drosophila Argonaute-2 Protein
Source: PLoS One. 2015 Mar 20;10(3):e0120205. doi: 10.1371/journal.pone.0120205 (PMC4368812; doi:10.1371/journal.pone.0120205)
Supplement: S1 Table — Sequences of mature miRNAs are bolded. (DOCX) [file pone.0120205.s001.docx]

**Supplementary Table 1. Primers and oligonucleotide sequences**

Sequences of mature miRNAs are bolded.

| **Primers** | **Sequences (5’ to 3’)** |
| --- | --- |
| NotI-ATG-mRFP | GCACGAGCGGCCGCATATGGCCTCCTCCGAGGACGTC |
| XhoI-Stop-mRFP | GCACGACTCGAGTTAGGCGCCGGTGGAGTGGCGG |
| **Oligonucleotides** | **Sequences (5’ to 3’)** |
| Pre-miRNA-1-white-Forward-EcoRI/SpHI | AAATTCATCGGGCCATTCGCTA**TTGGTTAATCAGCAGACCCTCG**TAGTTGTTTCCTAAACGAGGGTTTGTTGGTAGCTAAATAACGCATG |
| Pre-miRNA-1-white-Reverse-EcoRI/SpHI | CGTTATTTAGCTACCAACAAACCCTCGTTTAGGAAACAACTA**CGAGGGTCTGCTGATTAACCAA**TAGCGAATGGCCCGATG |
| Pre-miRNA-2-white-Forward-HindIII/ClaI | AGCTTAATCACAGCCTTTAATGTTGATTAACGTGGTTTTTCACAGTAAGTTAATATACCATATCTA**TTTTGAAAGGTCATGTTGGTCA**GTACCTAAAGTGCCTAACAT |
| Pre-miRNA 2-white-Reverse-HindIII/ClaI | CGATGTTAGGCACTTTAGGTAC**TGACCAACATGACCTTTCAAAA**TAGATATGGTATATTAACTTACTGTGAAAAACCACGTTAATCAACATTAAAGGCTGTGATTA |
| PM-target(x4)Forward-XhoI/XbaI | CACCCTCGAGTAATAGTGATGAAGGGCATCGACTTCAAGGATGAAGGGCATCGACTTCAAGGATGAAGGGCATCGACTTCAAGGATGAAGGGCATCGACTTCAAGGAGGTACCTCTAGAAG |
| PM-target(x4)Reverse-XhoI/XbaI | CTTCTAGAGGTACCTCCTTGAAGTCGATGCCCTTCATCCTTGAAGTCGATGCCCTTCATCCTTGAAGTCGATGCCCTTCATCCTTGAAGTCGATGCCCTTCATCACTATTACTCGAGGGTG |
| IM-target(x4)Forward-XhoI/XbaI | CACCCTCGAGTAATAGTGATGAAGGGCATCACTTTCAAGGATGAAGGGCATCACTTTCAAGGATGAAGGGCATCACTTTCAAGGATGAAGGGCATCACTTTCAAGGAGGTACCTCTAGAAG |
| IM-target(4x)Reverse-XhoI/XbaI | CTTCTAGAGGTACCTCCTTGAAAGTGATGCCCTTCATCCTTGAAAGTGATGCCCTTCATCCTTGAAAGTGATGCCCTTCATCCTTGAAAGTGATGCCCTTCATCACTATTACTCGAGGGTG |
